#  Modeling of the Full-Size 3D Structure of Human Chaperone Hsp70 and Study of Its Interdomain Interactions 

**Published:** 2010

**Authors:** К. А. Chernorizov, V.K. Švedas

**Affiliations:** Faculty of Bioengineering and Bioinformatics, Lomonosov Moscow State University; Belozersky Institute of Physicochemical Biology, Lomonosov Moscow State University

**Keywords:** chaperone, Hsp70, model, tertiary structure, ATPase and substrate-binding domains, molecular dynamics

## Abstract

Hsp70 is a chaperone protein that participates in the folding of de novo synthesized proteins, protection of the hydrophobic regions of denaturated proteins, the regulation of apoptosis, the immune response, and several other cellular processes. Despite the large number of publications devoted to the functioning and structure of Hsp70, a reliable full-size 3D structure of this protein remains currently unavailable. Several probable full-size models of human Hsp70 have been constructed based on the structures of individual domains and their components from different organisms and using molecular modeling methodology. The stability of the obtained structures was studied using molecular dynamics. As a result of such an analysis, the most adequate model was selected. The model was built on the basis of Hsp70 elements from*Bos Taurus*and*Caenorhabditis elegans*. Using the method of steered molecular dynamics, the key salt bridges responsible for the interdomain interactions were identified: Arg171: Glu516 and Arg416: Glu218. Based on the performed molecular modeling, the scheme of the mechanism triggering ATP hydrolysis and leading to the separation of ATPase and the substrate-binding domains was proposed.

##  INTRODUCTION 

**Fig. 1:**
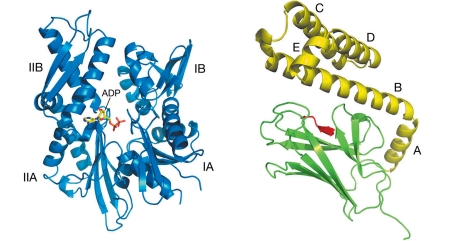
ATPase domain of human Hsp70 in complex with ADP (left; pdb ID 1HJO [6]) and the substrate-binding domain of Hsp70 prokaryotic homolog from *E.coli * – chaperone DnaK (right), consisting of αSBD (yellow), βSBD (green) in complex with substrate peptide (red); pdb ID 1DKZ) [7].

Hsp70 (Heat shock 70 kDa protein 1A/1B) is a two-domain ATP-dependent chaperone. Its function is to protect from aggregation the hydrophobic surfaces of proteins denaturated under stress and to promote their correct folding or direct them toward degradation. Proteins of this class are involved in the transport of peptides through the mitochondrial and cellular membranes. Depending on whether their localization is extracellular or intracellular, Hsp70s are involved, respectively, in the autoimmune labeling of tumor cells or in the protection of cells from stress and apoptosis [1, 2]. Hsp70 consists of two functionally distinct elements: the N-terminal ATPase domain hydrolyzes ATP to ADP with the formation of an inorganic phosphate; and the C-terminal substrate-binding domain binds hydrophobic peptides. The ATPase domain consists of two large subdomains, I and II, each of which is divided into the sections A and B (see Fig. 1). ATP binds at the bottom of the cleft located between the subdomains I and II. The ATPase domain also contains a binding site of the nucleotide exchange factor, which promotes the “recharging” of ADP to ATP by pulling the subdomains I and II apart from each other [3, 4].

The substrate-binding domain (SBD) consists of a β-sheet subdomain (βSBD), which binds the substrate peptide, and an α-helical “cap” (αSBD), formed by five α-helices A, B, C, D and E (see Fig. 1). Due to conformational changes mediated by ATP hydrolysis and the action of cochaperone Hsp40, the substrate-binding domain changes its state from a closed to an open one, while the “cap” regulates its accessibility for the hydrophobic sites of the target peptides [5].

Mediated by Hsp40, ATP hydrolysis and substrate binding lead to separation of the Hsp70 substrate-binding domain from the ATPase domain. As a result, they remain connected only by the hydrophobic linker. It is known that Hsp70 is capable of forming oligomers in solution. Most likely, this is due to the fact that the substrate-binding domain of Hsp70 is able to bind the hydrophobic linker of another Hsp70 molecule when its domains are separated [8, 9].

ATP hydrolysis is induced by the J-domain of cochaperone Hsp40. A study of the binding of Hsp70 and Hsp40 showed that Asp35 of yeast Hsp40 J-domain interacts with Arg171 of Hsp70. This leads to ATP hydrolysis and subsequent separation of Hsp70 domains from each other. The signal for ATP hydrolysis is assumed to pass through the chain J-domain of Hsp40 → Arg171 (interdomain linker of Hsp70) → Tyr371 (Hsp70 ATPase domain) → Ile181 (Hsp70 ATPase domain) [10-12]. Then, the substrate binds and domains separate from each other. The exact mechanism of this process remains unknown.

It is assumed that the structure of the substrate-binding domain of mammalian Hsp70 is similar to the structure of the analogous domain of its prokaryotic homolog DnaK (see Fig. 1) [7]. Although the structures of individual elements of the Hsp70 substrate-binding domain are known [11, 13], the full-size three-dimensional structure of mammalian Hsp70 is unknown. In this paper we attempt to construct a full-size three-dimensional structure of Hsp70 using molecular modeling methods. The structure is of interest not only for studying the interaction between ATPase and the substrate-binding domains of the chaperone, but also as a promising target for a search for anticancer agents. Because of their immunogenic properties, chaperones have been subjects of successful pharmacological studies [5, 14]. Establishing the plausible structure of human Hsp70 and the progress achieved in understanding the mechanism by which it functions could help to speed up research in the field of chaperone-associated cancer therapy strategies and reduce their cost.

##  EXPERIMENTAL 

Homology modeling was performed using the Swiss Model [15, 16]. The systems for molecular dynamics simulation were prepared in tleap (program from the AmberTools 1.2 package). Molecular dynamics simulations were performed in the software package Amber10 [17]. The protein molecule was put in a cubic cell, with the distance between the protein and the cell boundary not less than 12 Å. TIP3P water was used as a solvent. The integration step in all stages was 2 fs. Energy minimization was restricted to 5,000 steps, and the steepest descent algorithm was replaced by the conjugated gradient after 2,500 steps. The cutoff distance was 10 Å. The structures under study were equilibrated in four stages: energy minimization with position restraints on all atoms, energy minimization without restraints, heating and molecular dynamics simulation. Heating from 0 to 300 K was performed at a constant volume for 50 ps. Molecular dynamics was implemented at a constant pressure. The Langevin thermostat was used for temperature control. The same parameters were used in running steered molecular dynamics. In simulations of salt bridges, the disruption force constant was taken to be 20 kcal/mol•Å ^2^ . The visual analysis of three-dimensional structures was performed using VMD 1.8.6 [18].

##  RESULTS AND DISCUSSION 

**Modeling the full-size structure of Hsp70**

For the modeling, the following elements from the Protein Data Bank were used: substrate-binding domain of DnaK from *Escherichia coli* (1DKZ; Fig. 1); α-subdomain of Hsp70 substrate-binding domain from *Caenorhabditis elegans* (2P32 [19]); ATPase domain, βSBD and α-helix ‘A’ αSBD from bovine Hsc70 (1YUW). The latter structure describes the contact of the ATPase domain with the substrate-binding domain when the domains are rigidly coupled to each other.

**Model hHsp70_1dkz**

 Modeling was conducted in three steps: 

1. Based on a primary amino acid sequence of human Hsp70 (hHsp70) (PID: P08107) and a tertiary structure of its close homologue from *B. taurus* – Hsc70 (1YUW), a model was built describing the ATPase domain, βSBD and the helix ‘A’ of αSBD. The identity of the model with the template was 88.6%. To obtain a full-size structure, we had to construct the remaining part of the αSBD.

 2. To construct the αSBD the structure of the prokaryotic homolog of Hsp70, DnaK (1DKZ) was used as a template. In 1DKZ, the α-subdomain, in a complex with the β-subdomain, forms a full-size substrate-binding domain. The identity of the constructed structure with the template was 44.7%. 

**Fig. 2:**
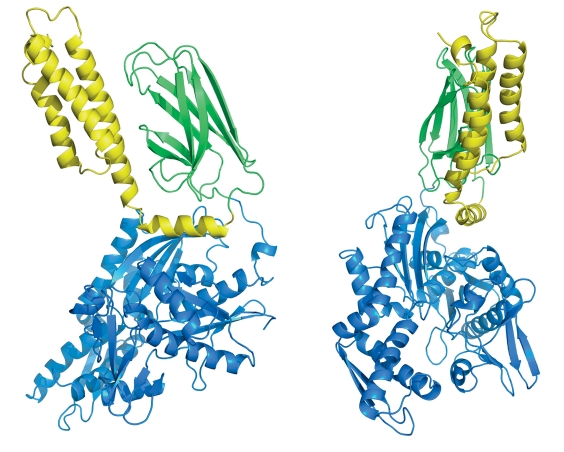
Model hHsp70_2p32 after molecular dynamics simulation. ATPase domain is colored blue, αSBD - yellow, βSBD – green.

 3. The resulting structures were superimposed by βSBD. The model was equilibrated using molecular dynamics simulation for 6.5 ns. 

In the resulting construct, the ATPase domain and βSBD were built by homology modeling with the close eukaryotic relative of Hsp70; and αSBD, by homology modeling with the prokaryotic chaperone DnaK from *E. coli* .

 An analysis of molecular dynamic trajectories showed that the spatial structures of the ATPase domain and βSBD were stable, while the segment of αSBD, formed by the α-helices ‘B’-’E’, was prone to unfolding. 

**Model hHsp70_2p32**

The structure of αSBD from nematode ( *Caenorhabditis elegans* ; PDB ID: 2P32) was taken as an alternative template for homologous modeling of the unstable segment. The identity of the resulting structure and the template was 63%. The created construction was superimposed on hHsp70_1dkz by the α-helix ‘B’, after which part of the α-helix ‘B’, as well as helices ‘C’, ‘D’ and ‘E’, was replaced by the corresponding elements constructed by homology modeling with the 2P32. The resulting model was equilibrated for 8.5 ns.

The model hHsp70_2p32 (after equilibration) is shown in Fig. 2. Helices ‘B’, ‘C’ and ‘D’ form a bundle similar in tertiary structure with 1DKZ; however, in contrast, the α-helix ‘E’ in hHsp70_2p32 is reduced. Based on an analysis of the molecular dynamic trajectory, the constructed structure showed high stability and was accepted as a working model (hereafter ‘hHsp70’) for further studies.

**Study of interdomain interactions in hHsp70**

 In the process of chaperone functioning, its domains diverge with a disruption of all noncovalent interactions. As a result, they remain connected only by a cross-domain linker. The process of domain separation was studied using the method of steered molecular dynamics. The salt bridges that are crucial in this process were identified. It was shown that inverse convergence of the domains is not dependent on conformational changes in the ATPase domain. 

**Disruption of salt bridge Arg171 : Glu516**

**Fig. 3:**
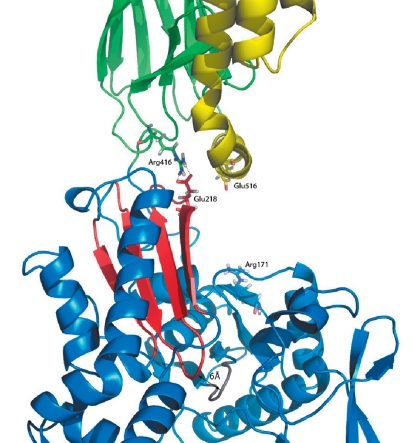
Rearrangement of the salt bridges during hHsp70 domains separation: on the right is the disrupted salt bridge Arg171: Glu516; on the left, the second bridge Arg416: Glu218 before simulation of its break. β-sheet including Glu218 and loop fixing the ATP is colored red. The second loop, which takes part in the fixation, is marked in gray.

According to [12], separation of the domains starts with (mediated by cochaperone Hsp40) disruption of the salt bridge Arg171: Glu516, which connects the α-helix ‘A’ of the substrate-binding domain with the subdomain IA from the ATPase domain. Modeling of this process was conducted using the method of steered molecular dynamics. The Cα-atoms of the residues were pulled apart by 10 Å. The initial distance between the atoms was 12 Å. The amount of energy spent on pulling the residues apart was 34.3 kcal / mol. It is worth noting that after the actual disruption of the salt bridge Arg171: Glu516, the average distance between loops of the ATPase domain fixing the β-and γ-phosphates of ATP decreased by 2 Å (from 8 to 6 Å; Fig. 3). In the native enzyme a similar movement, theoretically, could cause the hydrolysis of ATP. In such a case, the salt bridge Arg171: Glu516 protects the ATPase domain of hHsp70 from spontaneous ATP hydrolysis. The cochaperone disrupting this bond thus initiates the process.

**Fig. 4:**
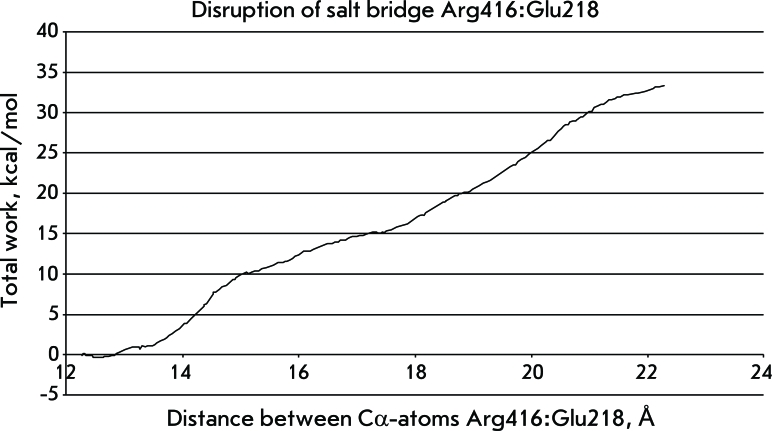
The work expended on the second stage of the simulation of ATPase and substrate-binding domains separation.

 The resulting structure was equilibrated for 1 ns. Signs of a restoration of the disrupted salt bridge Arg171: Glu516, as well as further domain separation, were not observed. 

**Disruption of salt bridge Arg416:Glu218**

 Disruption of the salt bridge Arg171: Glu516 led only to a partial divergence of the domains, but the final separation has not occurred. In this regard, it was interesting to identify the most stable noncovalent bonds whose disruption could lead to a complete separation of the hHsp70 domains. 

 Initially, the salt bridge Arg155: Glu523 connecting the α-helix ‘A’ with the subdomain IA was selected, but its disruption has not led to the domains’ separation and has shown no signs of positive development of the process. The negative result led to the conclusion that the salt bridge Arg416: Glu218 is the bond most likely to be key in domain divergence. It links one of the βSBD loops with the subdomain IIA of the ATPase domain. 

The initial distance between the Cα-atoms of the residues forming the bond was 12.2 Å; after simulation of its disruption, it was - 22.2 Å. The total amount of energy spent on the process was 33.4 kcal/mol. On a curve representing the process, a leap was observed: an increase in distance from 13.4 to 14.7 Å, accompanied by an immediate rupture of the salt bridge, required about 7 kcal/mol (Fig. 4).

**Fig. 5:**
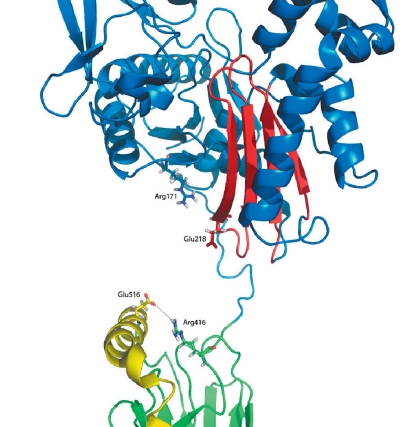
hHsp70 after the simulation of domains separation. The β-sheet, whose motion is accompanied by the disruption of the second salt bridge Glu218:Arg416, is colored red. The dotted line denotes the salt bridge Glu516:Arg416 formed after domain separation.

The energy spent on breaking the salt bridge was equal to the energy released in ATP hydrolysis; it could, therefore, be assumed that breakage of the macroergic ATP bond in the ATPase domain (presumably caused in the disruption of the salt bridge Arg171: Glu516) is required to break the salt bridge Arg416: Glu218. Glu218 is part of the β-sheet (formed by the amino acid residues 192 – 226 and 332 – 338; Fig. 5), the second β-turn of which immediately fixes the β- and γ-phosphates of ATP. The first β-turn is involved in the contact of ATPase with substrate-binding domains. Considering the fact that the β-sheet is a rigid structure, its displacement, mediated by ATP hydrolysis, can cause a breakage of the second bond required for the divergence of domains.

 As expected, simulation of the breakage of the bond Arg416: Glu218 has led to a complete separation of domains. The resulting structure was equilibrated for 1.5 ns, during which no significant changes in the structure were observed. 

An interesting fact was observed in the simulation of domain separation: the residues Arg416 and Glu516 liberated from salt bridges later formed a new salt bridge with each other (see Fig. 5). Formation of a similar salt bridge is sterically possible also between Arg171 and Glu218.

 Based on these data we can draw a conclusion on the important role of interdomain salt bridges in the functioning of hHsp70: the salt bridges Arg171: Glu516 and Arg416: Glu218 connect ATPase and substrate-binding domains, but as the domains diverge from each other the partners are rearranged and can form the salt bridges Arg171: Glu218 and Arg416: Glu516, which stabilize the new structural state of the chaperone. 

**Simulation of separation of subdomains I and II**

 In the process of nucleotide exchange, ATPase and substrate-binding domains converge back, but the force involved in this process has yet to be established. A stochastic nature of domain convergence seems unlikely. Because nucleotide exchange occurs while domains are separated and is accompanied by the interaction of the ATPase domain with the nucleotide exchange factor, it is reasonable to assume that pulling the subdomains I and II apart by the nucleotide exchange factor, theoretically, can affect the convergence of ATPase and the substrate-binding domains. We conducted a simulation of the process. In simulating pulling apart subdomain I from subdomain II, points of application of force were taken to be the nitrogen atoms belonging to residues Thr14 and Gly203, directly interacting with ATP / ADP. The initial distance between them was 7.3 Å; the final one, 14.3 Å. Despite the theoretical possibility that nucleotide exchange affects the convergence of domains, we obtained a predictable result: change in the distance between the subdomains I and II did not affect the interaction of the domains with each other. This result suggests that the convergence of domains is most likely mediated by some protein which may be another chaperone hHsp70. Theoretically, it can bind the hydrophobic linker released in domain divergence as a substrate and thus bring the diverged domains closer. However, this hypothesis requires further verification. 

##  CONCLUSIONS 

 Basing on research, the following scheme of hHsp70 functioning can be suggested: at the interaction with the J-domain of cochaperone Hsp40, the salt bridge Arg171: Glu516 is broken. This leads to a convergence of the loops fixing ATP and promotes ATP hydrolysis. ATP hydrolysis, in turn, is accompanied by a shift of the β-sheet containing one of the loops fixing ATP and a shift of the residue Glu218 that is located in the site of interdomain contact, which leads to the disruption of the salt bridge Arg416: Glu218. Disruption of the salt bridge Arg416: Glu218 results in separation of the ATPase domain from the substrate-binding domain. Arg416 forms a salt bridge with Glu516 that belongs to the substrate-binding domain. Arg171, in turn, forms a new salt bridge with Glu218. Thus, regrouping of the salt bridges that previously connected ATPase and the substrate-binding domains of hHsp70 occurs. 

 The role of the nucleotide exchange factor in the functioning of hHsp70, apparently, is not only in performing the exchange of ADP for a new molecule of ATP, but also in the fixation of the subdomains I and II in separated states, which is accompanied by the convergence of ATPase and substrate-binding domains with formation of the salt bridge Arg171: Glu516 that can be figuratively described as “cocking the hammer” for the next round of ATP hydrolysis. The “finger” that can pull the trigger is the J-domain of cochaperone Hsp40. 

An equilibrated model of human Hsp70 can be found in the PMDB depositary [20, 21] (PMDB id: PM0076412).
